# Using the Neutrophil-to-Lymphocyte Ratio and Platelet-to-Lymphocyte Ratio as Prognostic Markers for Obstructive Sleep Apnea: A Systematic Review and Meta-Analysis of Observational Studies

**DOI:** 10.7759/cureus.72539

**Published:** 2024-10-28

**Authors:** Mahmoud Mohamed Attia, Mohammad Alameen Qasim, Hamzeh Samer Alhamwi, Mahmoud Ali, Ebtihal Abdelhay Alsharief, Judy Sobhy Ali, Mohamed Elmasry, Ahmed Elgebaly, Eshak I Bahbah

**Affiliations:** 1 Faculty of Medicine, Al-Azhar University, Damietta, EGY; 2 Department of Internal Medicine, MedDots Academy, Cairo, EGY; 3 Department of Clinical Pharmacy, Al-Emamin Al-Kadhimeen City Teaching Hospital, Baghdad, IRQ; 4 Faculty of Pharmacy, Philadelphia University, Amman, JOR; 5 Faculty of Pharmacy, Cairo University, Cairo, EGY; 6 Faculty of Medicine, Cairo University, Cairo, EGY; 7 Faculty of Medicine, Alexandria University, Alexandria, EGY; 8 Smart Health Centre, University of East London, London, GBR

**Keywords:** diagnostic biomarkers, neutrophil-to-lymphocyte ratio, obstructive sleep apnea, platelet-to-lymphocyte ratio, prognostic biomarkers

## Abstract

Obstructive sleep apnea (OSA) is a chronic condition characterized by the partial or complete collapse of the airways during sleep, leading to hypoxemia (reduced oxygen flow to organs) and disrupted sleep. This study aims to establish robust evidence for the relationship between the neutrophil-to-lymphocyte ratio (NLR) and platelet-to-lymphocyte ratio (PLR) and the severity and prognosis of OSA. A systematic review and meta-analysis were conducted using PubMed, Cochrane Central Register of Controlled Trials, Scopus, Medline, and Web of Science to gather all relevant articles. Twenty-six eligible articles were included in the study. The pooled outcomes indicated that the NLR and PLR were significantly higher in patients with OSA compared to controls. Subgroup analyses based on the severity of OSA showed that differences in the NLR were more pronounced in patients with severe OSA. Moreover, meta-regression analysis revealed that variations in mean age, BMI, and male proportion did not significantly affect the differences in the NLR and PLR. OSA has a significant impact on NLR and PLR levels, making them useful markers for detecting the inflammatory status in OSA patients.

## Introduction and background

Obstructive sleep apnea (OSA) is a chronic disorder characterized by the partial or total collapse of the airways during sleep, leading to hypoxemia and sleep disturbances. The prevalence of OSA is notably higher in older men, particularly those between 50 and 70 years of age, compared to women of the same age group [1]. In children, OSA is often associated with factors such as tonsil enlargement and adenoid grade, with a higher prevalence in boys compared to girls, at a ratio of 3:1 [2]. Among middle-aged adults, OSA is common, affecting approximately 4% of men and 2% of women [3].

OSA is also potentiated and predisposed by obesity since overweight patients (BMI>25) form about 60% of the obstructive sleep apnea syndrome population and obese (BMI>30) make up at least 30% of the population [4]. The recurrence of airway collapse leads to sleep fragmentation and excessive daytime sleep. Many diseases are reported to be associated with OSA such as cardiovascular hypertension, dyslipidemia, diabetes mellitus, and an increase in the mortality rate [5,6].

OSA is a chronic inflammatory disorder and many systemic inflammatory markers participated in the pathogenesis of it. So, we can use the systemic inflammatory markers as tools to assess the OSA and help us in its management [7,8]. Among these markers, the neutrophil-to-lymphocyte ratio (NLR) and platelet-to-lymphocyte ratio (PLR) are notable. The NLR and PLR can be obtained from routine blood tests which makes them low-cost and available tools for inflammatory prognosis for many chronic diseases [9,10]. Several studies evaluate the relationship between the NLR and PLR and the severity and prognosis of OSA. Some of them showed there was a significant elevation in the NLR and PLR in patients with OSA, while other studies showed there was no relationship between them [4,11-35]. This conflict of results may be due to the difference in the sample size, study design, and criteria of the study population. Therefore, we conducted this systematic review and meta-analysis to evaluate the relationship between the NLR and PLR and the severity and prognosis of OSA.

## Review

Methods

While reporting this systematic review and meta-analysis, we followed the Preferred Reporting Items for Systematic Reviews and Meta-Analyses (PRISMA) and Meta-analysis of Observational Studies in Epidemiology (MOOSE) statement guidelines [36,37].

Search Criteria

A systematic review was conducted by searching MEDLINE via PubMed, Scopus, Web of Science, and Cochrane Central Register of Controlled Trials (CENTRAL). The search strategy utilized the following keywords: (("sleep apnea syndromes" OR "obstructive sleep apnea" OR "sleep apnea" OR OSA OR "sleep-disordered breathing") AND ("neutrophil to lymphocyte" OR "neutrophil lymphocyte" OR "neutrophil-to-lymphocyte" OR NLR OR "platelet to lymphocyte" OR "platelet lymphocyte" OR PLR)).

Eligibility Criteria and Study Selection

This study included all studies meeting the following criteria: observational studies published in English, involving patients diagnosed with OSA characterized by recurrent episodes of nocturnal hypoxemia, who underwent full-night polysomnography in a laboratory or other diagnostic tests. The studies must have assessed differential complete blood count tests, with the NLR and PLR calculated by dividing the absolute values of neutrophils by lymphocytes, and platelets by lymphocytes, respectively. The severity of OSA cases was evaluated based on age, comorbid conditions, or the apnea-hypopnea index (AHI), which classifies OSA severity as follows: normal or non-OSA (simple snoring, AHI < 5), mild (AHI 5-14), moderate (AHI 15-29), and severe (AHI ≥ 30). Conversely, review articles, animal studies, and studies that were published in languages other than English were excluded. Additionally, studies that did not assess at least one of the inflammatory markers (NLR or PLR), or that did not investigate OSA separately from other clinical conditions, were also excluded. Two independent reviewers screened titles and abstracts to identify potentially eligible studies. Full-text articles were then reviewed against the predefined inclusion and exclusion criteria. Any disagreements between the reviewers were resolved through discussion or, if necessary, by consulting a third reviewer.

Quality Assessment

The risk of bias and quality of each study were assessed using the Newcastle-Ottawa Scale (NOS). The NOS evaluates studies based on three main domains: 1) Selection: This domain assesses the adequacy of the case definition, representativeness of the cases, selection of controls, and definition of controls. A study can receive a maximum of four stars in this domain. 2) Comparability: This domain evaluates the comparability of cases and controls based on the design or analysis. It examines whether the study controlled for the most important factors and additional factors. A study can receive a maximum of two stars in this domain. 3) Exposure: This domain assesses the ascertainment of exposure, whether the same method of ascertainment was used for cases and controls, and the non-response rate. A study can receive a maximum of three stars in this domain. Each study was independently assessed by two reviewers, and any disagreements were resolved through discussion or consultation with a third reviewer.

Data Extraction

Two reviewers independently extracted the data from the included studies using a predefined extraction form. Any discrepancies were resolved through discussion or by consulting a third reviewer. Data extracted from eligible studies included the first author's last name, year of publication, country where the study was conducted, study design, group subclassification within the primary groups, number of group members, age in years, number and percentage of male participants, body mass index (BMI) calculated as kilograms per square meter, NLR, PLR, and the main findings of the study.

Data Analysis

Data analysis was conducted using Review Manager (RevMan) version 5.4 (Cochrane Collaboration, UK). A random-effects model was employed for the meta-analysis to account for potential clinical heterogeneity among the included studies. Statistical heterogeneity was assessed using the Chi-squared test and the I² statistic, with a p-value of less than 0.10 for the chi-squared test or an I² value greater than 50% indicating substantial heterogeneity. Results were reported as risk ratios (RRs) for dichotomous outcomes and mean differences (MDs) for continuous outcomes, both with 95% confidence intervals (CIs). A p-value of less than 0.05 was considered statistically significant. Due to limited data, no subgroup analysis was planned. Meta-regression analysis was performed to identify the impact of certain factors on the effect size. A subgroup analysis based on the disease severity and the use of continuous positive airway pressure (CPAP) was conducted.

Results 

Results of Literature Search

**Figure 1:**
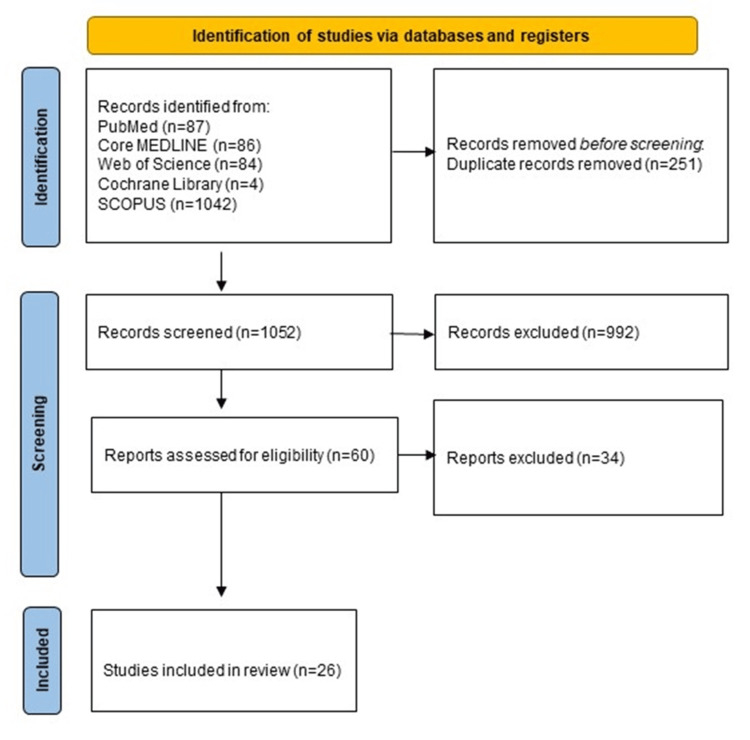
PRISMA flow diagram. PRISMA: Preferred Reporting Items for Systematic Reviews and Meta-Analyses

After conducting a literature search across five databases, we identified 1,303 articles. Following the removal of duplicates (n = 251), 1,052 articles remained for title and abstract screening. Of these, 60 articles were selected for full-text screening, and ultimately, 26 studies met the criteria for inclusion in this systematic review and meta-analysis [4,11-35], as shown in Figure 1.

The characteristics of the included studies are summarized in Table 1.

**Table 1:** Characteristics of included studies. TTT: Treatment, OSA: Obstructive sleep apnea, BMI: Body mass index, NOS: Newcastle-Ottawa scale, NLR: Neutrophil-to-lymphocyte ratio, PLR: Platelet-to-lymphocyte ratio, AHI: Apnea-hypopnea index, CPAP: Continuous positive airway pressure, OSAS: Obstructive sleep apnea syndrome *Median (IQR)

Study ID	Country	Study design	Groups	Sample size	Age, year (mean (SD))	Gender, male (n (%))	BMI, Kg/m^2^ (mean (SD))	NOS	Main findings
Al-Halawani 2020 [30]	USA	Cohort study	Before CPAP TTT	109	58.10 (14.01)	106 (97.00)	34.12 (6.43)	4	There is a clear relationship between NLR and severity of OSA
			After CPAP TTT						
Anjum 2019 [23]	Pakistan	Cross-sectional	Control	63	45.22 (2.57)	36 (57.10)	23.90 (2.66)	5	The PLR increased in people with OSA
			Mild OSA (AHI= 5-15)	60	49.85 (2.11)	46 (76.70)	24.03 (2.74)		
			Moderate OSA (AHI= 16-30)	67	50.37 (2.28)	60 (89.60)	27.00 (3.03)		
			Severe OSA (AHI > 30)	90	52.34 (2.60)	74 (82.20)	26.54 (2.66)		
Atlintas 2015 [25]	USA and Turkey	Cohort study	Control	80	47.30 (10.80)	56 (70.00)	32.50 (7.80)	9	The NLR in severe OSA patients was significantly higher than in mild or moderate OSA patients and healthy controls.
			Mild OSA (AHI= 5-15)	163	48.90 (12.80)	90 (73.00)	32.40 (7.30)		
			Moderate OSA (AHI= 16-30)	158	48.30 (10.80)	111 (70.00)	32.60 (8.40)		
			Severe OSA (AHI > 30)	160	48.90 (10.50)	110 (69.00)	34.30 (7.50)		
Bozkuş 2018 [4]	Turkey	Cohort study	Control	42	44.02 (11.35)	25 (59.50)	23.31 (1.62)	7	The NLR ratio rises as the obesity grade increases
			Normal (AHI>5 / BMI<25) OSA	36	42.00 (11.24)	20 (45.60)	23.34 (1.54)		
			Overweight (AHI>5 / BMI 25-30) OSA	38	43.00 (7.16)	21 (55.30)	27.82 (1.24)		
			Obesity (AHI>5 / BMI >30) OSA	39	43.69 (7.42)	25 (64.10)	36.29 (2.29)		
Duger 2020 [35]	Germany	Cohort study	Control	86	45.10 (3.20)	54 (63.00)	32.30 (5.90)	6	The NLR was elevated relatively in patients with OSA
			Mild OSA (AHI= 5-15)	30	44.00 (9.40)	18 (60.00)	30.80 (5.90)		
			Moderate OSA (AHI= 16-30)	24	43.00 (8.40)	16 (67.00)	32.80 (6.90)		
			Severe OSA (AHI > 30)	32	46.70 (12.00)	20 (63.00)	31.00 (5.40)		
Erdim 2017 [33]	Turkey	Cohort study	Non-OSA (AHI < 1)	38	11.76 (1.38)	14 (36.80)	NA	5	The NLR and PLR were significantly higher in children whose AHI was ≥5 than in children from the other groups.
			OSA (AHI < 5)	24	11.63 (1.17)	14 (58.30)			
			OSA (AHI < 5) combined (control)	62	11.71 (1.29)	28 (66.70)			
			OSA (AHI> 5)	21	11.71 (1.38)	7 (33.30)			
Fan 2018 [32]	China	Cohort study	Control	135	46.30 (12.04)	135 (100.00)	24.00 (3.32)	3	The NLR and PLR have no significant impact as an inflammatory marker
			Mild OSA (AHI= 5-15)	185	44.90 (11.10)	185 (100.00)	25.40 (2.87)		
			Moderate OSA (AHI= 16-30)	171	46.00 (11.17)	171 (100.00)	26.70 (3.22)		
			Severe OSA (AHI > 30)	596	43.50 (10.93)	596 (100.00)	29.10 (7.93)		
Friššcic 2022 [29]	Croatia	Cohort study	OSA before CPAP	37	53.00 (10.00)	29 (78.00)	34.40 (6.10)	8	In patients with severe OSA, the NLR demonstrated the beneficial effects of CPAP therapy on the degree of inflammation.
			OSA after CPAP						
Günbatar 2015 [14]	Turkey	Cohort study	Control	26	44.70 (10.40)	21 (80.00)	27.15 (5.00)	5	The NLR and PLR are good predictors of OSA severity
			Moderate OSA (AHI= 16-30)	22	49.30 (10.60)	16 (72.70)	32.40 (4.80)		
			Severe OSA (AHI > 30)	63	51.02 (11.10)	50 (79.36)	33.50 (5.90)		
Khaliq 2019 [22]	Pakistan	Cross-sectional	Obese with OSA	32	20-40 years	32 (100.00)	> 25 Kg/m2	6	Between the two study groups, the NLR is not significantly different.
			Obese without OSA (control)	32		32 (100.00)			
Kim 2023 [17]	Korea	Cross-sectional	Control	95	38.4	70 (73.70)	23.8	6	Analysis showed no significant correlation between the PLR and NLR values and the OSA severity groups.
			Mild OSA (AHI= 5-15)	204	46.9	174 (85.30)	25.1		
			Moderate OSA (AHI= 16-30)	286	48	243 (85.00)	26		
			Severe OSA (AHI > 30)	517	47.1	471 (93.00)	28.4		
Korkmaz 2015 [21]	Turkey	Cohort study	Control	40	43.30 (11.14)	14 (35.00)	29.27	7	The severity of the amount of systemic inflammation in OSAS patients cannot be predicted by the NLR.
			Mild OSA (AHI= 5-15)	27	44.96 (9.25)	18 (66.67)	29.15		
			Moderate OSA (AHI= 16-30)	37	47.24 (9.12)	21 (56.76)	31.97		
			Severe OSA (AHI > 30)	43	49.35 (9.79)	26 (60.47)	32.60		
Koseoglu 2014 [19]	Turkey	Cohort study	Control	57	43.50 (11.20)	23 (40.00)	29.00 (4.80)	7	The PLR is strongly associated with the severity of OSA
			Mild OSA (AHI= 5-15)	93	51.10 (8.60)	58 (62.00)	30.20 (4.80)		
			Moderate OSA (AHI= 16-30)	82	51.10 (10.90)	62 (76.00)	32.40 (6.40)		
			Severe OSA (AHI > 30)	192	51.60 (10.60)	139 (72.00)	35.30 (7.20)		
Koseoglu 2015 [27]	Turkey	Case-control	Control	48	43.08 (8.88)	29 (60.40)	27.06 (3.76)	7	There is a relationship between the NLR and PLR with the severity of OSA
			Mild OSA (AHI= 5-15)	67	47.25 (10.14)	167 (70.76)	30.67 (4.60)		
			Moderate OSA (AHI= 16-30)	61			30.67 (4.60)		
			Severe OSA (AHI > 30)	108			30.67 (4.60)		
Koseoglu 2020 [31]	Turkey	Cohort study	Control	29	41.80 (7.80)	19 (65.52)	27.30 (3.40)	5	There is no relationship between the NLR and PLR and the severity of OSA
			Mild OSA (AHI= 5-15)	30	45.50 (10.10)	19 (63.33)	28.20 (4.60)		
			Moderate OSA (AHI= 16-30)	33	47.00 (9.50)	20 (60.61)	29.90 (3.90)		
			Severe OSA (AHI > 30)	49	47.20 (9.90)	31 (63.27)	30.70 (4.50)		
Kum 2020 [15]	Turkey	Cross-sectional	Control	46	38.20 (14.73)	27 (58.70)	24.91 (4.19)	6	The severity of OSA may not be determined by NLR levels.
			Mild OSA (AHI= 5-15)	26	41.13 (14.08)	43 (64.18)	28.96 (4.05)		
			Moderate OSA (AHI= 16-30)	26			29.65 (3.28)		
			Severe OSA (AHI > 30)	15			30.38 (4.22)		
Kıvanc 2017 [24]	Turkey	Case-control	Control	52	41.00 (12.00)	35 (67.00)	29.00 (4.30)	7	The NLR cannot show the negative effects of comorbidities linked to OSA.
			Mild OSA (AHI= 5-15)	53	43.00 (10.00)	44 (83.00)	29.00 (4.50)		
			Moderate OSA (AHI= 16-30)	65	50.00 (11.00)	47 (72.00)	31.30 (4.30)		
			Severe OSA (AHI > 30)	130	51.00 (11.00)	101 (78.00)	34.00 (6.00)		
Oyama 2015 [12]	Japan	Cohort study	Control	5	50.00 (14.60)	2 (40.00)	21.90 (3.00)	7	A high NLR is strongly associated with the severity of OSA, CPAP therapy successfully reduces endothelial dysfunction and inflammation in OSA patients
			Mild OSA (AHI= 5-15)	14	61.80 (17.30)	9 (64.00)	22.40 (3.40)		
			Moderate OSA (AHI= 16-30)	26	63.80 (9.40)	18 (69.00)	26.30 (5.20)		
			Severe OSA (AHI > 30)	50	59.30 (13.10)	43 (86.00)	28.70 (5.30)		
			Severe OSA (AHI>30) before CPAP	29	62.30 (9.50)	24 (83.00)	27.30 (3.60)		
			Severe OSA (AHI>30) after CPAP for 3 months						
Özdemir 2019 [20]	Turkey	Cohort study	Before CPAP TTT	29	46.00 (10.11)	18 (62.10)	34.85 (5.75)	6	The PLR and NLR are not clear predictors for the effect of CPAP TTT on systematic inflammation in patients with OSA
			After CPAP TTT				35.51 (5.88)		
Sunbul 2015 [18]	Turkey	Case-control	Control	65	48.70 (10.20)	42 (64.62)	26.90 (4.90)	6	Patients with OSA had a significantly higher NLR than controls
			Mild OSA (AHI= 5-15)	20	47.40 (8.50)	12 (60.00)	33.40 (8.80)		
			Moderate OSA (AHI= 16-30)	35	50.80 (9.60)	26 (74.29)	33.10 (5.10)		
			Severe OSA (AHI > 30)	75	50.10 (10.30)	53 (70.67)	35.30 (8.50)		
Ulusoy 2019 [34]	Turkey	Cohort study	Control	30	41.00 (13.5) *	16 (53.00)	24.35 (1.60)	7	NLR and PLR values of the patient group (before and after PAP treatment) were significantly lower than the control group.
			Moderate OSA (AHI 16-30)	15	47.00 (20.8) *	13 (86.70)	28.36 (4.80)		
			Severe OSA (AHI>30)	21		16 (76.20)	31.48 (6.30)		
			Moderate OSA (AHI 16-30) after CPAP	15		13 (86.70)	28.36 (4.80)		
			Severe OSA (AHI>30) after CPAP	21		16 (76.20)	31.48 (6.30)		
Uygur 2016 [13]	Turkey	Cohort study	Control	118	50.30 (11.70)	61 (51.69)	29.40 (7.80)	7	OSAS patients had higher NLRs than controls.
			Mild OSA (AHI= 5-15)	57	53.70 (10.80)	36 (63.16)	30.80 (5.70)		
			Moderate OSA (AHI= 16-30)	53	51.80 (12.10)	30 (56.60)	31.60 (8.10)		
			Severe OSA (AHI > 30)	61	54.50 (12.70)	39 (63.93)	32.10 (7.10)		
Wuttiumporn 2017 [28]	Thailand	Non-randomized Clinical trial	Severe OSA (AHI>30) before CPAP	20	43.50 (10.50)	6 (30.00)	25.70 (3.70)	NA	CPAP shows clinical benefits in treating severe OSA patients by improving the inflammatory biomarker profile
			Severe OSA (AHI>30) after 90 days CPAP						
			Severe OSA (AHI>30) after 180 days CPAP						
Yenigun 2015 [26]	Turkey	Cohort study	Control	38	48.08 (8.82)	20 (53.63)	30.54 (6.16)	8	The NLR has a positive correlation with OSAS severity
			Mild OSA (AHI= 5-15)	34	46.75 (8.05)	24 (70.59)	33.97 (6.79)		
			Moderate OSA (AHI= 16-30)	30	53.64 (12.60)	14 (46.67)	33.53 (6.66)		
			Severe OSA (AHI > 30)	34	52.94 (12.21)	18 (52.94)	36.15 (6.63)		
			Severe OSA (AHI>30) treated with CPAP for 3 months	34	50.75 (13.09)	20 (59.82)	32.25 (3.67)		
Zorlu 2021 [16]	Turkey	Case-control	Control	54	53.10 (11.20)	26 (48.15)	NA	6	The NLR and PLR could be an indicator of OSAS, but they don't have to increase in parallel with OSAS severity.
			Mild OSA (AHI= 5-15)	41	53.40 (11.00)	21 (51.22)			
			Moderate OSA (AHI= 16-30)	54	54.50 (10.30)	32 (59.26)			
			Severe OSA (AHI > 30)	58	55.20 (14.50)	42 (72.41)			
Zota 2022 [11]	Romania	Case-control	Control	31	49.55 (14.01)	16 (51.60)	32.11 (5.16)	7	There is no significant correlation between PLR, NLR, and OSA, or OSA severity, or even AHI
			Mild OSA (AHI= 5-15)	33	54.06 (15.37)	20 (60.60)	32.34 (5.44)		
			Moderate OSA (AHI= 16-30)	22	57.68 (9.18)	16 (72.60)	32.65 (6.16)		
			Severe OSA (AHI > 30)	37	58.49 (9.49)	26 (70.30)	35.41 (5.63)		

Difference in the NLR Between OSA Patients and Controls

Twenty studies [4,11-18,21,22,24-27,32-35] were included in the analysis comparing the NLR between OSA patients and controls. The pooled random-effects model indicated that the NLR was significantly higher in OSA patients than in the control group (SMD = 0.38, 95% CI: 0.17 to 0.59; p = 0.0004). However, substantial heterogeneity was present in the pooled data (Tau² = 0.56; Chi² = 820.7, P < 0.00001; I² = 94%).

Subgroup Analysis According to Disease Severity

**Figure 2:**
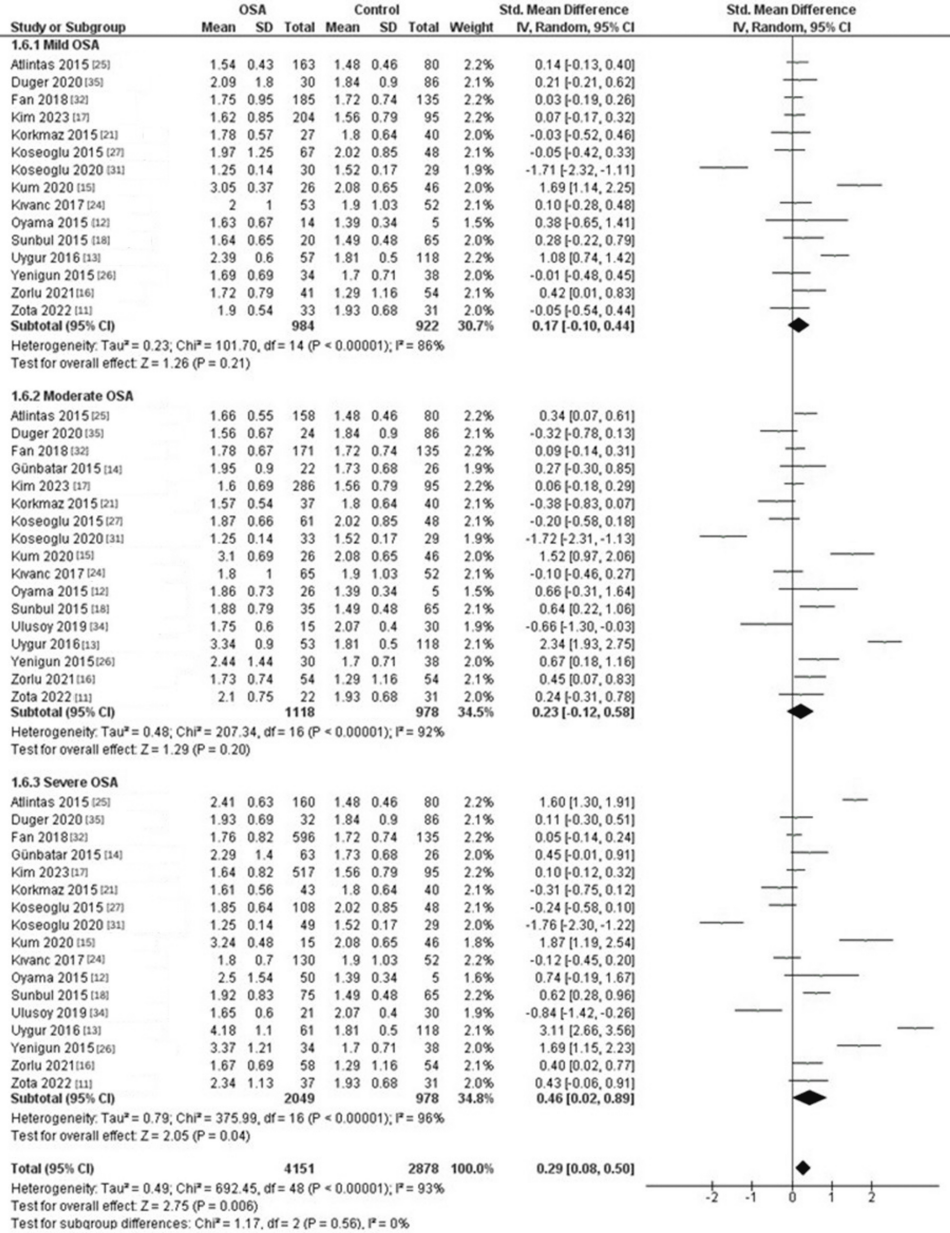
Forest plot of subgroup analysis of the NLR between OSA patients and controls according to disease severity. OSA: Obstructive sleep apnea, NLR, Neutrophil-to-lymphocyte ratio, SD: Standard deviation, CI: Confidence interval, IV: Inverse variance

Subgroup analysis based on disease severity revealed that NLR differences in mild and moderate OSA were not statistically significant compared to controls (mild OSA: SMD = 0.18, 95% CI: -0.1 to 0.45; p = 0.21; moderate OSA: SMD = 0.23, 95% CI: -0.12 to 0.59; p = 0.19). However, patients with severe OSA had a significantly higher NLR than controls (SMD = 0.46, 95% CI: 0.02 to 0.9; p = 0.04, Figure 2).

Difference in the NLR in OSA Patients Before and After CPAP

**Figure 3:**
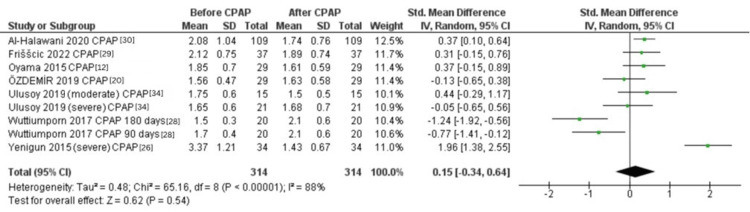
Forest plot of the effect of CPAP on the NLR in OSA patients. OSA: Obstructive sleep apnea, NLR, Neutrophil-to-lymphocyte ratio, SD: Standard deviation, CI: Confidence interval, IV: Inverse variance, CPAP: Continuous positive airway pressure

Seven studies [12,20,26,28-30,34] examined the NLR before and after CPAP treatment. The pooled random-effects model showed no significant difference in NLR before and after CPAP (SMD = 0.15, 95% CI: -0.34 to 0.64; p = 0.54, Figure 3). The data were highly heterogeneous (Tau² = 0.48; Chi² = 65.16, P < 0.00001; I² = 88%).

Difference in the PLR Between OSA Patients and Controls

**Figure 4:**
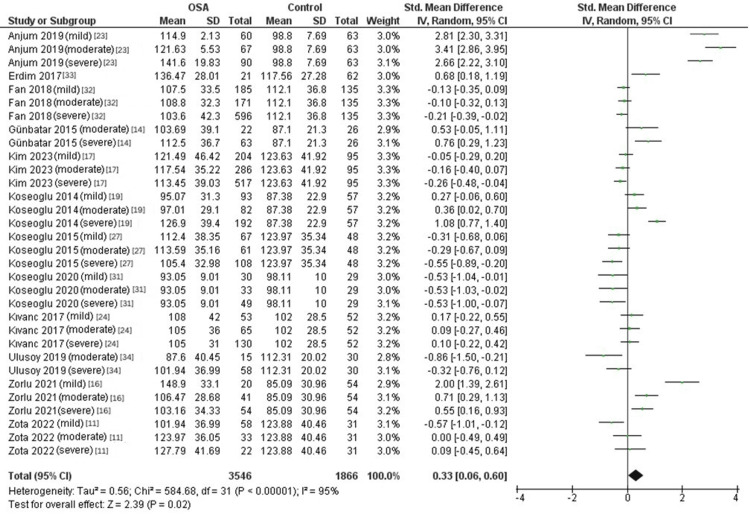
Forest plot of random-effects model SMD of the PLR between OSA patients and controls. OSA: Obstructive sleep apnea, NLR, Neutrophil-to-lymphocyte ratio, SD: Standard deviation, CI: Confidence interval, IV: Inverse variance, CPAP: Continuous positive airway pressure, SMD: Standardized mean difference

Twelve studies [11,14,16,17,19,23,24,27,31-34] were analyzed to compare the PLR between OSA patients and controls. The pooled random-effects model indicated that the PLR was significantly higher in OSA patients than in the control group (SMD = 0.33, 95% CI: 0.06 to 0.6; p = 0.02, Figure 4). Substantial heterogeneity was observed in the pooled data (Tau² = 0.56; Chi² = 584.68, P < 0.00001; I² = 95%).

Subgroup Analysis According to Disease Severity

**Figure 5:**
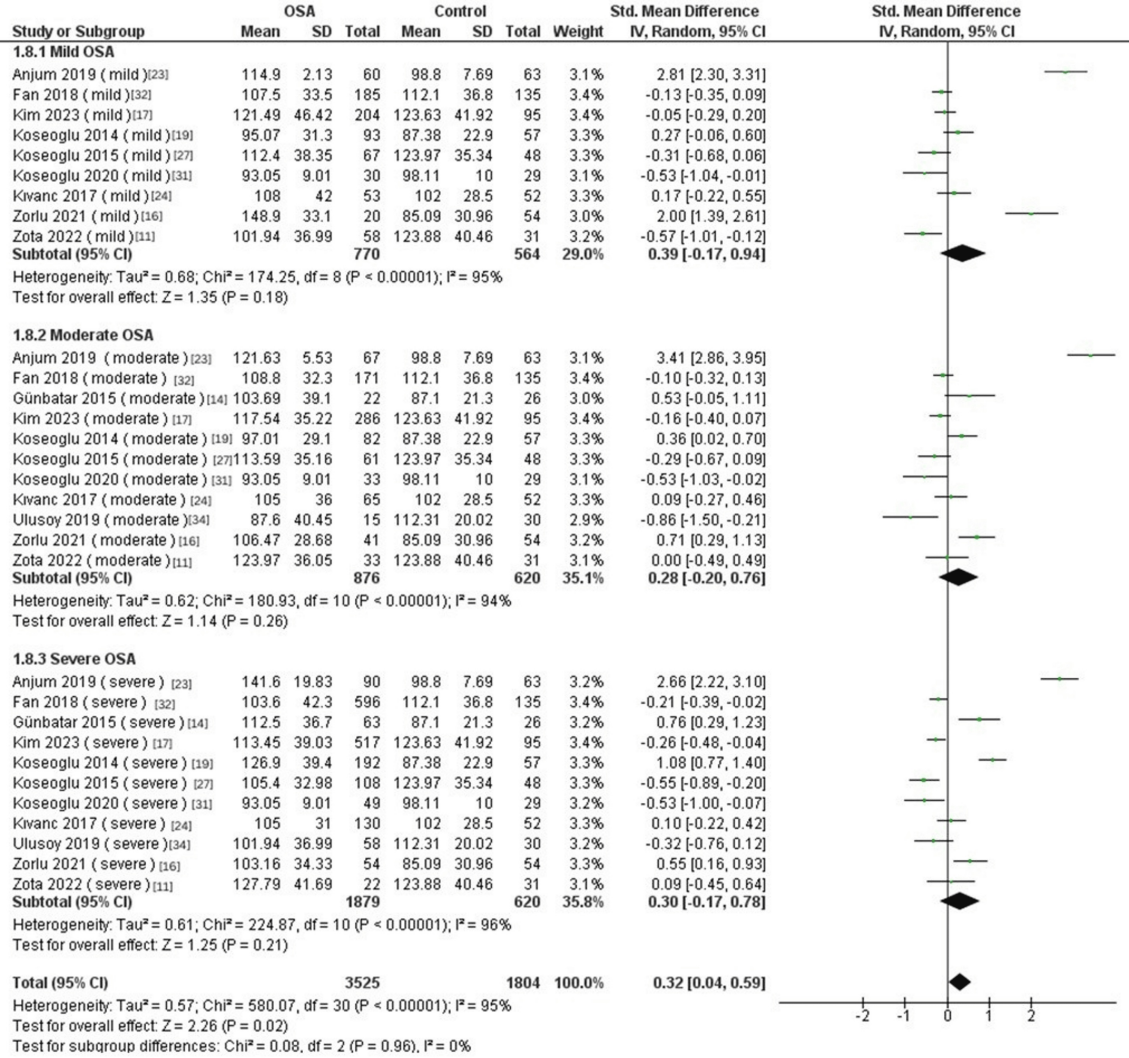
Forest plot of subgroup analysis of PLR and OSA according to disease severity. OSA: Obstructive sleep apnea, PLR: Platelet-to-lymphocyte ratio, SD: Standard deviation, CI: Confidence interval, IV: Inverse variance, CPAP: Continuous positive airway pressure

Subgroup analysis based on disease severity revealed no significant differences in the PLR between mild, moderate, and severe OSA compared to controls (mild OSA: SMD = 0.39, 95% CI: -0.17 to 0.94; p = 0.18; moderate OSA: SMD = 0.28, 95% CI: -0.2 to 0.76; p = 0.26; severe OSA: SMD = 0.3, 95% CI: -0.17 to 0.78; p = 0.21, Figure 5).

Difference in the PLR in OSA Patients Before and After CPAP

**Figure 6:**
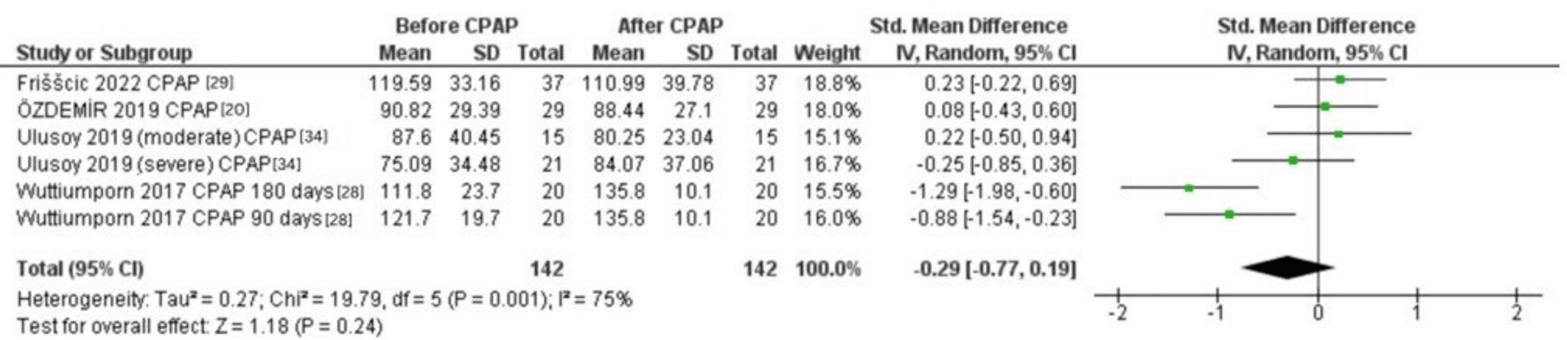
Forest plot of the effect of CPAP on the PLR. OSA: Obstructive sleep apnea, PLR: Platelet-to-lymphocyte ratio, SD: Standard deviation, CI: Confidence interval, IV: Inverse variance, CPAP: Continuous positive airway pressure

Four studies [20,28,29,34] examined the PLR before and after CPAP treatment. The pooled random-effects model showed no significant difference in the PLR before and after CPAP (SMD = -0.29, 95% CI: -0.77 to 0.19; p = 0.24, Figure 6). The data were substantially heterogeneous (Tau² = 0.27; Chi² = 19.79, P = 0.001; I² = 75%).

Meta-Regression Analysis

A meta-regression analysis was conducted to assess the impact of age (years), BMI (kg/m²), and the proportion of male patients (%) on differences in the NLR and PLR between OSA patients and controls. The analysis revealed that variations in age, BMI, and the proportion of male patients (%) did not significantly influence NLR and PLR differences (Table 2).

**Table 2:** Meta-regression. SE: Standard error, NLR: Neutrophil-to-lymphocyte ratio, PLR: Platelet-to-lymphocyte ratio

Factor	Parameter	β	95% CI	SE	p-value
Age	NLR	0.011	-0.035 to 0.058	0.024	0.63
	PLR	0.016	-0.033 to 0.065	0.025	0.52
BMI	NLR	0.035	-0.089 to 0.159	0.063	0.58
	PLR	-0.147	-0.299 to 0.005	0.078	0.059
Male%	NLR	1.048	-1.208 to 3.304	1.151	0.363
	PLR	-1.172	-3.727 to 1.382	1.303	0.368

Discussion 

This systematic review and meta-analysis showed that OSA patients have a significantly higher NLR and PLR compared to controls. This suggests that OSA may have an impact on the systemic inflammatory response, as reflected by the elevated NLR [38]. Neutrophils are key players in the innate immune response, while lymphocytes are involved in adaptive immunity. An increased NLR may indicate an imbalance in the immune system, with a relative increase in the neutrophil count and a decrease in the lymphocyte count [39]. This imbalance could be attributed to chronic intermittent hypoxia and sleep fragmentation, which are characteristic features of OSA.

In the context of OSA, the elevated NLR may serve as an indicator of increased systemic inflammation and could potentially be used for risk stratification and monitoring of disease progression [40]. Further studies are warranted to explore the clinical utility of NLR in OSA management and its potential as a therapeutic target. The finding of a higher PLR in OSA patients is consistent with previous studies that have reported an association between OSA and increased systemic inflammation. Platelets and lymphocytes play crucial roles in the inflammatory response, and an elevated PLR indicates a potential imbalance between pro-inflammatory and anti-inflammatory processes [41]. The present study provides valuable insights into the potential role of PLR as a biomarker for systemic inflammation in OSA. The significant difference in the PLR between OSA patients and the control group suggests that PLR may serve as a useful clinical indicator for assessing the inflammatory status in OSA patients. Monitoring PLR levels could help identify individuals at higher risk of developing OSA-related complications and guide the implementation of targeted interventions [38].

Our findings revealed interesting results when considering the disease severity in a subgroup analysis. We observed that the difference in NLR between mild and moderate OSA patients was comparable to the control group, while severe OSA patients had significantly higher NLR compared to the control group. These findings may imply that the inflammatory burden associated with mild and moderate OSA is not substantial enough to significantly affect the NLR. In contrast, our data revealed a significant difference in NLR between severe OSA patients and the control group. This indicates that severe OSA is associated with an increased NLR, suggesting a heightened inflammatory response in these patients. The elevated NLR in severe OSA patients may be indicative of a more pronounced systemic inflammation, which could be attributed to the repetitive hypoxia-reoxygenation cycles and oxidative stress associated with severe OSA [30]. These findings align with previous studies that have demonstrated a link between severe OSA and increased inflammation markers.

Despite the lack of a significant difference in the NLR before and after CPAP therapy, it is worth noting that individual studies within the analysis reported conflicting results. Some studies demonstrated a significant decrease in the NLR following CPAP treatment [38], indicating a potential beneficial effect on systemic inflammation. On the other hand, other studies reported no significant change in the NLR after CPAP therapy [11]. These discrepancies may be due to variations in study populations, sample sizes, and other methodological differences. It was hypothesized that CPAP therapy, by improving sleep quality and reducing intermittent hypoxia, would lead to a decrease in NLR levels. However, the results of this meta-analysis suggest that CPAP may not have a significant impact on systemic inflammation as measured by the NLR.

Our meta-regression analysis did not find any significant effects of the age of patients on the NLR and PLR. This finding is consistent with previous studies that have reported no association between age and NLR or PLR in OSA patients [11]. It is important to note that age is a complex factor influenced by various physiological and pathological processes, and its relationship with inflammatory markers such as NLR and PLR may be influenced by other confounding factors not accounted for in our analysis.

Our analysis did not find any significant effects of BMI on the NLR and PLR. Although obesity is a known risk factor for OSA, our results suggest that BMI alone may not be a strong predictor of NLR and PLR alterations in this context. This finding is in line with previous studies that have reported inconsistent associations between BMI and NLR or PLR in OSA patients [2]. It is possible that other factors related to obesity, such as adipose tissue inflammation or metabolic dysfunction, may have a more direct impact on NLR and PLR changes in OSA patients.

The proportion of male patients did not show any significant effect on the NLR and PLR. This finding contradicts previous studies that have reported higher NLR and PLR values in male OSA patients compared to females [35]. The lack of significance in our analysis may be attributed to the limited number of studies included in our meta-regression, as well as the potential influence of other unmeasured confounding factors. Further research with larger sample sizes and more comprehensive adjustments for confounders is needed to elucidate the role of gender in NLR and PLR alterations in OSA patients.

Our study holds several strengths that contribute to the robustness of its findings. We conducted a comprehensive systematic search in multiple databases minimizing the publication bias and manual screening to ensure all relevant studies were included. Also, all included studies were at low risk of bias in terms of randomization, allocation and blinding. However, certain limitations warrant consideration in the interpretation of our results. First, we included only English language studies. Another one in which our analysis had some unresolved heterogeneity due to variations in study populations, participant characteristics, sample sizes, and other methodological differences.

## Conclusions

This systematic review and meta-analysis demonstrate that the NLR and PLR are significantly elevated in patients with OSA, particularly in those with severe cases. These findings suggest that the NLR and PLR could serve as valuable biomarkers for detecting systemic inflammation in OSA, offering potential utility in assessing disease severity and guiding management strategies. While CPAP therapy effectively addresses the mechanical symptoms of OSA, its impact on reducing systemic inflammation as measured by these markers appears limited. Further research is needed to confirm these biomarkers' clinical value and their role in improving patient outcomes.
